# Prevalence of Circadian Rhythm Sleep-Wake Disorders and Associated Factors in Euthymic Patients with Bipolar Disorder

**DOI:** 10.1371/journal.pone.0159578

**Published:** 2016-07-21

**Authors:** Yoshikazu Takaesu, Yuichi Inoue, Akiko Murakoshi, Yoko Komada, Ayano Otsuka, Kunihiro Futenma, Takeshi Inoue

**Affiliations:** 1 Department of Psychiatry, Tokyo Medical University, 6-7-1 Nishishinjuku, Shinjuku-ku, Tokyo, 160–0023, Japan; 2 Department of Somnology, Tokyo Medical University, 6-7-1 Nishishinjuku, Shinjuku-ku, Tokyo, 160–0023, Japan; RIKEN Brain Science Institution, JAPAN

## Abstract

Recent studies have suggested that there are certain pathophysiological relationships between bipolar disorder (BD) and circadian rhythm dysfunction. However, apparently no studies have clarified the prevalence of circadian rhythm sleep-wake disorders (CRSWD) in patients with BD. This study was set out to investigate the prevalence of CRSWD and associated factors in patients with BD. One hundred four euthymic BD outpatients participated in this study. The subjects were asked to answer questionnaires including demographic variables, clinical course of BD, and family history of psychiatric disorders and suicide. Severity of BD was assessed by the Montgomery-Åsberg Depression Rating Scale and Young Mania Rating Scale. CRSWD was diagnosed by clinical interview, together with sleep logs, according to the International Classification of Sleep Disorders, third edition (ICSD-3). Thirty-five subjects (32.4%) met the criteria for CRSWD. The age at the time of investigation and that at the onset of BD were both lower in the CRSWD group than in the non-CRSWD group. The rates of family history of psychiatric disorders and suicide in the CRSWD group were higher than those in the non-CRSWD group. Multiple logistic regression analysis revealed that the presence of CRSWD was significantly associated with younger onset age of BD and family history of suicide. The prevalence of CRSWD could be quite high in BD patients. Younger onset age of BD and family history of suicide were associated with presence of CRSWD in BD patients.

## Introduction

The circadian system regulates daily rhythms of physiology and behavior, such as the sleep-wake cycle, core body temperature, hormonal secretion, and mood [1]. Recent studies have suggested that circadian rhythms play a critical role in emotional dysregulation in bipolar disorder (BD) [2–5]. Changes in the sleep-wake cycle, such as decreased need for sleep, insomnia, or hypersomnia, are parts of the diagnostic criteria for BD in the Diagnostic and Statistical Manual of Mental Disorders, Fifth Edition (DSM-5) [6]. Typically, during the manic phase, there is a reduced need for sleep, whereas during the depressive phase, individuals suffer from insomnia or hypersomnia [4,7]. Moreover, even during the euthymic period, disruption of sleep-wake cycle has been reported in previous studies [8,9]. Of note, the Systematic Treatment Enhancement Program for Bipolar Disorder (STEP-BD) study showed that an increase in sleep variability was associated with greater depressive and manic symptoms [10]. The circadian rhythm hypothesis of BD emphasizes that instability of the circadian rhythm indicates core vulnerability of BD and that disturbance in circadian rhythm is a critical factor for the onset and exacerbation of BD [2,4].

A recent paper reviewed treatment of BD from the viewpoint of circadian rhythms [11]. Several studies have indicated that the mood stabilizer lithium affects circadian rhythms through glycogen synthase kinase 3β, which is a central regulator of the circadian clock [12–14]. Social rhythm stabilization by interpersonal and social rhythm therapy is also effective in reducing relapse of BD [15]. Additionally, a recent study showed that adjunctive ramelteon, which has an effect in synchronizing the circadian clock to the day-night cycle, was effective for preventing relapse of BD [16]. These clinical findings suggest that disrupted circadian rhythm may be involved in the pathological mechanism of BD [17].

Circadian rhythm sleep-wake disorder (CRSWD) is defined by the following criterion, namely, the disorder is caused by alterations of the circadian time-keeping system, its entrainment mechanisms, or a misalignment of the endogenous circadian rhythm to the external environment [18]. A previous study reported that melatonin secretion in euthymic BD patients was suppressed and its peak was delayed compared to those in patients with remitted major depression and normal controls [19]. Another study revealed that BD patients are more likely to have the eveningness chronotype, suggesting a circadian phase delay in these patients [20]. Moreover, there were some common circadian gene polymorphisms among patients with BD, delayed sleep-wake phase disorder, and non-24-hour sleep-wake disorder, which are sub-categories of CRSWD [21]. These study results raise the possibility that BD patients are highly vulnerable for having CRSWD.

However, there apparently has been no systematic study focusing on the prevalence of CRSWD in BD patients and the clinical characteristics of BD patients with CRSWD comorbidity. Therefore, we set a cross-sectional study to investigate the prevalence and factors associated with the presence of CRSWD in patients with BD.

## Materials and Methods

### Subjects

This study was approved by the ethics committee of Tokyo Medical University and conducted after obtaining written informed consent from the subject patients. The consecutive bipolar participants were recruited from patients who visited the outpatient clinic of the Neuropsychiatric Department in Tokyo Medical University Hospital from August 2014 to January 2015. There were 127 patients 18 to 75 years old who met the criteria for bipolar I or II disorder according to DSM-5. Participants were eligible if they were euthymic as defined by the Young Mania Rating Scale (YMRS <7 points) and Montgomery-Åsberg Depression Rating Scale (MADRS <13 points) for at least 8 weeks prior to the investigation [22]. Exclusion criteria included the following: (a) patients were in affective episodes, (b) shift worker, (c) ongoing alcohol or substance abuse, (d) suicidal risk, (e) hospitalized in the previous 8 weeks, or (f) had visual impairments, who were all more likely to have CRSWD [23,24]. Twelve patients refused to participate in the study and 11 patients met the exclusion criteria. As a result, 104 euthymic BD patients were included in the present study.

### Assessments

The subjects were asked to answer questionnaires, including demographic variables, clinical descriptive variables of bipolar disorder and circadian rhythm-related sleep problems, and family history of either psychiatric disorders or suicide. The current mood status was assessed using the MADRS [25] and YMRS [26]. Assessment of subjective sleep disturbance was conducted by using the Pittsburgh Sleep Quality Index (PSQI) [27,28]. The diagnoses of CRSWD were made with clinical interview by a board-certified sleep specialist physician together with the results of sleep logs for more than 4 weeks according to the International Classification of Sleep Disorders, third edition (ICSD-3) [18]. The subjects who met the criteria for CRSWD were additionally divided into sub-categories of CRSWD according to the ICSD-3 [18].

### Statistical Analyses

The Mann-Whitney U test and chi-square test were used for the comparison of descriptive variables between the subjects who met the criteria for CRSWD (CRSWD group) and those who did not meet the criteria for CRSWD (non-CRSWD group). The chi-square test or Fisher’s exact test was used for the comparison of kinds of medications for the treatment of mood disorders (mood stabilizers, antipsychotics, antidepressants, and benzodiazepines) between the two groups. The Mann-Whitney U test was also used for the comparison of the PSQI score, YMRS score, and MADRS score between the two groups.

Thereafter, factors associated with the presence of CRSWD were examined with the aid of a series of logistic regression analyses with independent variables (sex, age at the time of investigation, age at the onset of BD, duration of illness, living alone or not, educational background, being employed or not, type of BD diagnosis, presence or absence of family history of suicide or psychiatric disorders, MADRS score, YMRS score, and PSQI scores). All variables were initially examined in univariate models. To control for confounding factors and to determine the main correlates, we then performed multivariate logistic regression analyses for all variables that showed significant correlations in univariate models. SPSS version 11.5.1J software for Windows (SPSS Inc., Tokyo) was used for the above statistical analyses. A *p*-value of less than 0.05 was considered to indicate a statistically significant difference.

## Results

**Fig 1:**
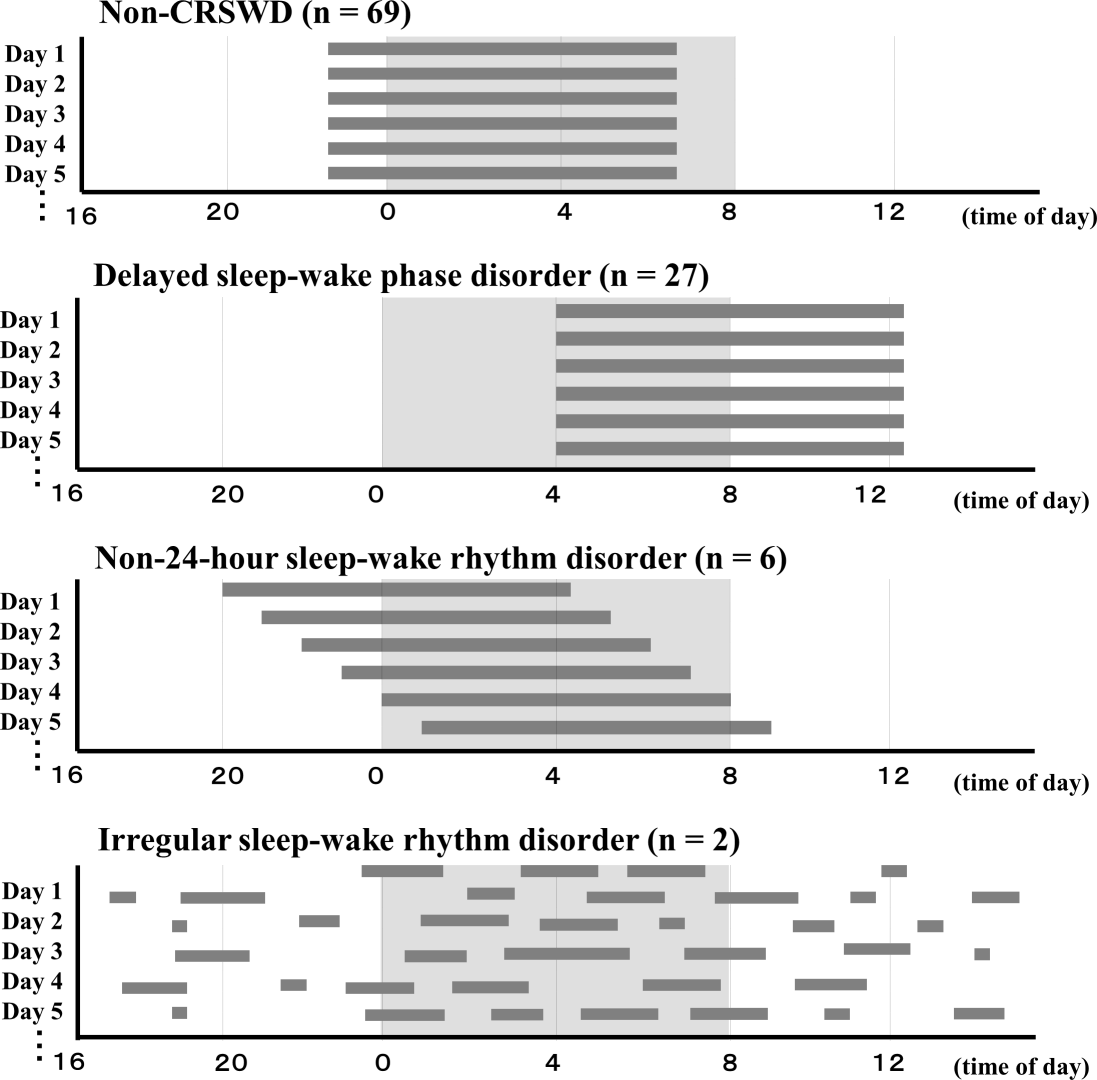
Sub-categories of CRSWD in BD patients. A total of 27 patients met the criteria for delayed sleep-wake phase disorder, 6 patients met the criteria for non-24-hour sleep-wake rhythm disorder, and 2 patients met the criteria for irregular sleep-wake rhythm disorder. CRSWD = circadian rhythm sleep-wake disorder. BD = bipolar disorder.

Of the total 104 subjects, there were 35 subjects (32.4%) who met the criteria for CRSWD. According to the sub-categories of CRSWD in ICSD-3, 27 patients met the criteria for delayed sleep-wake phase disorder, which is characterized by a significant delay in the phase of the major sleep episode in relation to the desired or required sleep time and wake-up time [29,30] (Fig 1). Six patients met the criteria for non-24-hour sleep-wake rhythm disorder, which is characterized by symptoms of insomnia or excessive sleepiness that occur because the intrinsic circadian pacemaker is not entrained to a 24-hour light/dark cycle [29,30] (Fig 1). All these 6 patients with non-24-hour sleep-wake rhythm disorder were unemployed and experienced a transition from delayed sleep-wake phase disorder. Two patients met the criteria for irregular sleep-wake rhythm disorder, which is characterized by lack of a clearly defined circadian rhythm of sleep and wake [29,30] (Fig 1). There was no patient who met the criteria for advanced sleep-wake phase disorder, which is characterized by a stable advance (earlier timing) of the major sleep episode, such that habitual sleep onset and offset occur typically two or more hours prior to required or desired times [29,30].

In the CRSWD group, 12 subjects out of 35 total subjects (34.3%) answered “onset of their sleep disturbance was prior to that of mood disturbance”, 8 subjects (22.8%) answered “onset of mood disturbance was prior to that of CRSD”, and 15 subjects (42.9%) answered “Both of these disturbances occurred almost at the same time”.

Both age at the time of investigation (*p* = 0.004) and onset age (*p*<0.001) of bipolar disorder in the CRSWD group were significantly younger than those in the non-CRSWD group. The rates of family history of both suicide and psychiatric disorders in the CRSWD group were significantly higher than those in the non-CRSWD group. No significant differences in any other descriptive variables were found between the two groups (Table 1).

**Table 1:** Comparison of the demographics between the two groups.

	BD patients with CRSWD (N = 35)	BD patients without CRSWD (N = 69)	*p*-value
Age at the time of investigation (years)	41.7±15.2 (range: 23–73)	48.9±14.4 (range: 20–74)	0.004
Sex (male/female)	15/20	28/41	0.836
Onset age of BD (years)	23.0±11.3	33.0±13.1	<0.001
Duration of illness (years)	18.7±16.8	16.4±16.0	0.506
Living alone (yes/no)	25/10	57/12	0.210
College graduate (yes/no)	18/17	35/34	1.000
Employed (yes/no)	11/24	20/49	0.823
Family history of psychiatric disorders (yes/no)	19/16	22/47	0.034
Family history of suicide (yes/no)	9/26	4/65	0.009
Type of BD (I/II)	14/21	27/42	1.000
MADRS score (points)	4.6±4.1	3.9±4.5	0.217
YMRS score (points)	1.9±1.9	1.8±2.1	0.623
PSQI score (points)	8.3±4.0	7.3±3.8	0.210

BD, bipolar disorder; CRSWD, circadian rhythm sleep-wake disorder; MADRS, Montgomery-Åsberg Depression Rating Scale; YMRS, Young Mania Rating Scale; PSQI, Pittsburgh Sleep Quality Index

Values are expressed as mean ± SD. The Mann-Whitney U test was used for the comparison of continuous variables between the 2 groups. The chi-square test or Fisher’s exact test was used for the comparison of categorical variables between the 2 groups.

Concerning medication, no significant differences in taking any mood stabilizers, antipsychotics, antidepressants, or benzodiazepines were found between the two groups (Table 2).

**Table 2:** Comparison of the medications between the two groups.

	BD patients with CRSWD (N = 35)	BD patients without CRSWD (N = 69)	*p*-value
Lithium (yes/no)	12/23	18/51	0.492
Lamotrigine (yes/no)	17/18	28/41	0.531
Valproate (yes/no)	4/31	13/56	0.410
Carbamazepine (yes/no)	4/31	3/66	0.359
Olanzapine (yes/no)	1/34	8/61	0.267
Quetiapine (yes/no)	2/33	9/60	0.327
Risperidone (yes/no)	2/33	2/67	0.359
Antidepressants (yes/no)	5/30	10/59	1.000
Benzodiazepines (yes/no)	26/9	58/11	0.294

BD, bipolar disorder; CRSWD, circadian rhythm sleep-wake disorder

The chi-square test or Fisher’s exact test was used for the comparison of categorical variables between the 2 groups.

Univariate logistic regression analysis showed that age at the time of investigation (*p* = 0.016), onset age of BD (*p* = 0.001), and family history of suicide (*p* = 0.007) and psychiatric disease (*p* = 0.029) were significantly associated with the presence of CRSWD. Multiple logistic regression analysis revealed that onset age of BD (*p* = 0.002) and family history of suicide (*p* = 0.038) were significantly associated with the presence of CRSWD in the subject BD patients (Table 3). When the cut-off value of probability of predicting CRSWD was 0.5, the sensitivity was 48.6%, specificity was 92.8%, and predictive accuracy was 77.9% in this regression analysis model.

**Table 3:** Multivariate logistic regression analysis of the associated factors for CRSWD (N = 104).

	Univariate odds ratio (95% CI)	*p*-value	Multivariate odds ratio (95% CI)	*p*-value
Age at the time of investigation (years)	0.96 (0.93–0.99)	0.016	0.97 (0.93–1.00)	0.068
Onset age of BD (years)	0.93 (0.90–0.97)	0.001	0.94 (0.90–0.98)	0.002
Family history of psychiatric disorders (yes/no)	2.54 (1.10–5.85)	0.029	1.99 (0.78–5.08)	0.150
Family history of suicide (yes/no)	5.63 (1.59–19.88)	0.007	4.19 (1.05–16.27)	0.038

BD, bipolar disorder; CRSWD, circadian rhythm sleep-wake disorder

Only variables at *p* < 0.05 in the univariate models are shown in this table.

## Discussion

To our best knowledge, this is the first study to investigate the prevalence of CRSWD in BD patients in clinical settings. The prevalence of CRSWD in our BD patients (32.4%) seems to be higher compared with that in the general population, which was reported to be 0.13% in the general Japanese population [31] and 0.17% in the general Norwegian population [32]. However, we did not compare it with the prevalence of CRSWD in age- and sex-matched controls or with a population with other psychiatric disorders. Therefore, we could not draw the definite conclusion that the prevalence of CRSWD in BD was significantly higher than that in the general population from this study. One study indicated that BD patients showed a distinctive distribution of chronotype compared with normal controls, schizophrenia patients, and schizoaffective disorder patients [33].

Recently, a new evaluation tool for biological rhythm dysfunction in BD patients was developed (BRIAN: Biological Rhythm Interview of Assessment in Neuropsychiatry) [34]. Several studies revealed the relationship between biological rhythm dysfunction and BD by using BRIAN [35–37]. These studies indicated that biological rhythm dysfunction in BD was more severe than that in healthy controls and in patients with major depressive disorder [36]. Moreover, severity of biological rhythm dysfunction was associated with severity of depressive symptoms and poor psychosocial functioning in BD [37]. Supporting the idea that CRSWD may be more prevalent in BD, the results of these studies indicated a robust pathophysiological relationship between circadian rhythm dysfunction and BD.

In the results of the present study, the most frequent subcategory of CRSWD was delayed sleep-wake phase disorder (27/35, 77.1%). This result is in line with the previous studies showing delayed peak of melatonin secretion and eveningness chronotype predominance in BD patients [19,20,38]. Moreover, a recent study revealed that eveningness chronotype was associated with greater sleep-wake disturbance, worse quality of life, and more dysfunctional sleep-related cognition and behaviors [39]. Interestingly, the result of this study revealed that the rate of individuals having non-24-hour sleep-wake rhythm disorder, which is prevalent in nearly half of totally blind individuals, but thought to be rare in sighted individuals [23,24], was remarkably high in the CRSWD affected patients (6/35, 17.1%) even though no blind individuals participated in this study. The etiology of non-24-hour sleep-wake rhythm disorder and the pathophysiological association between this disorder and bipolar disorder have not been well documented in previous studies. In this regard, only Hayakawa et al. reported that more than half of their 57 patients with non-24-hour sleep-wake rhythm disorders were diagnosed as having psychiatric disorders before or after the onset of the disorder [40]. However, their psychiatric diagnoses were mainly major depression and no patient was diagnosed with bipolar disorder. The inconsistency between the present study and the previous report is unclear. However, considering that in approximately 20% of patients receiving an initial diagnosis of depression the diagnosis is changed to bipolar disorder within 20 years [41], one possible explanation for this inconsistency is that the age of the patients who were diagnosed as having major depression in the study of Hayakawa et al. were too young (26.2±8.5 years) to make accurate psychiatric diagnosis for bipolar-unipolar distinction compared with the age of the patients in our study (46.7±14.6 years). Previous studies have indicated that patients with non-24-hour sleep-wake rhythm disorders are likely to have a preceding period with delayed sleep-wake phase disorders and the symptoms of delayed sleep-wake phase disorders change to non-24-hour sleep-wake rhythm disorders possibly due to the loss of social zeitgebers [40,42]; these are in line with the results of the present study. Taking these factors into consideration, delayed circadian rhythm can be one of the characteristic features of euthymic BD, especially in young-onset cases with high likelihood of transition to free running of circadian rhythm in this kind of patient.

Although the causal relationship between BD and CRSWD cannot be determined in this cross-sectional study, about one-third of subjects with CRSWD answered “onset of sleep disturbance was prior to that of mood disturbance”. Previous studies have suggested that sleep changes preceded mood dysregulation in BD patients [2,43,44]. Perlman et al. reported that sleep disturbance predicted relapse of depressive symptoms through the 6-month follow-up on BD patients [45]. Although it is difficult to draw a conclusion about the relationship between sleep disturbance and mood dysregulation in BD (2), at least it can be said that sleep disturbances, including CRSWD, are associated with the primary pathophysiology of BD.

Interestingly, the results of our study showed that the presence of CRSWD was significantly associated with younger onset age of BD and family history of suicide in the subject patients. Dysregulation of sleep-wake pattern has been reported to be an early marker of BD in familial high-risk individuals [46]. Moreover, BD patients with younger onset age were reported to have more eveningness chronotype, which is closely related to the occurrence of delayed sleep-wake phase disorder [33]. Concerning genetic factors, some circadian genes have been suggested to be related to the pathogenesis of BD [12–14,47–50]. Among these, the CLOCK gene has been found to be associated with clinical features of BD, such as the increase in motor activity in the evening, delayed sleep phase [48], and recurrent episodes of mood dysfunction [51]. Interestingly, CLOCK mutant mice showed mania-like behavior that is reversed by treatment with the mood stabilizer lithium [52]. Taking these into consideration, common genetic backgrounds between CRSWD and BD may exist.

This study has several limitations. First, because this study was conducted as a single-center study, the sample size was relatively small and subjects might not be representative of general bipolar patients. A multi-center study with a larger sample size will be needed to confirm our findings. Second, because we did not use age- and sex-matched controls, we could not draw any conclusion about the difference in the prevalence between BD patients and the general population. Third, we did not use reliable objective measures of circadian rhythm, such as actigraphy or measurement of endogenous melatonin secretion, which are recommended in the ICSD-3 criteria [18]. We also did not use the valuable assessment of biological rhythm BRIAN as mentioned above. Fourth, it was possible that BD patients with concomitant CRSWD suffered from more difficulties than those without CRSWD, with the consequence of their higher consultation rate and apparent high prevalence of CRSWD in BD patients. Fifth, although there were no significant differences in the rate of prescriptions between the two groups with our relatively small sample size, it is possible that some medications, particularly medications having sedative effects, might have contributed to the association between CRSWD and BD. Sixth, we analyzed three different types of CRSWD (delayed sleep-wake phase disorder, non-24-hour sleep-wake rhythm disorder, and irregular sleep-wake rhythm disorder) as a single group because of the small numbers of patients with each disorder. Although these three disorders are likely to convert to each other and are thought to have a similar biological mechanism [40,42], this heterogeneity might have contributed to the results of this study. Seventh, the ratios of employed/unemployed were similar between the subjects with/without CRSWD in this study. We investigated the employment status of patients only with a simple question “Do you have an occupation now?”. However, we should have asked them about details of their employment status (e.g. regular employment, part-time job, or housewife), because circadian rhythm is strongly affected by social factors. If we investigated details of employment status, there might have been some significant differences in employment status between the two groups.

In conclusion, the results of this study revealed that CRSWD is frequently comorbid with BD. Younger onset age of BD and family history of suicide were associated with the presence of CRSWD in the subject BD patients. Therefore, it can be speculated that there are underlying common pathophysiological backgrounds between BD and CRSWD. In the future, a longitudinal follow-up study with a larger sample size and comparison of the prevalence of CRSWD with controls would be needed to confirm the findings of the present study.
